# An investigation of herpes simplex virus promoter activity compatible with latency establishment reveals VP16-independent activation of immediate-early promoters in sensory neurones

**DOI:** 10.1099/vir.0.034728-0

**Published:** 2011-11

**Authors:** João T. Proença, Heather M. Coleman, Michael P. Nicoll, Viv Connor, Christopher M. Preston, Jane Arthur, Stacey Efstathiou

**Affiliations:** 1 Division of Virology, Department of Pathology, University of Cambridge, Tennis Court Road, Cambridge CB2 1QP, UK; 2 MRC–University of Glasgow Centre for Virus Research, 8 Church Street, Glasgow G11 5JR, Scotland, UK; 3 Microbiology and Infectious Diseases Laboratories, Institute of Medical and Veterinary Science, Frome Road, Adelaide 5000, Australia

## Abstract

Herpes simplex virus (HSV) type-1 establishes lifelong latency in sensory neurones and it is widely assumed that latency is the consequence of a failure to initiate virus immediate-early (IE) gene expression. However, using a Cre reporter mouse system in conjunction with Cre-expressing HSV-1 recombinants we have previously shown that activation of the IE ICP0 promoter can precede latency establishment in at least 30 % of latently infected cells. During productive infection of non-neuronal cells, IE promoter activation is largely dependent on the transactivator VP16 a late structural component of the virion. Of significance, VP16 has recently been shown to exhibit altered regulation in neurones; where its *de novo* synthesis is necessary for IE gene expression during both lytic infection and reactivation from latency. In the current study, we utilized the Cre reporter mouse model system to characterize the full extent of viral promoter activity compatible with cell survival and latency establishment. In contrast to the high frequency activation of representative IE promoters prior to latency establishment, cell marking using a virus recombinant expressing Cre under VP16 promoter control was very inefficient. Furthermore, infection of neuronal cultures with VP16 mutants reveals a strong VP16 requirement for IE promoter activity in non-neuronal cells, but not sensory neurones. We conclude that only IE promoter activation can efficiently precede latency establishment and that this activation is likely to occur through a VP16-independent mechanism.

## Introduction

Primary infection with herpes simplex virus (HSV) results in lifelong latency within sensory neurones followed by periodic episodes of virus reactivation. During latency, the virus genome is largely transcriptionally repressed and the only viral transcripts readily detected comprise the latency-associated transcripts (LATs) (reviewed by Efstathiou & Preston, 2005; Wagner & Bloom, 1997). The LATs are encoded within the repeats flanking the unique-long region of the virus genome. The primary 8.3 kb LAT transcript termed minor LAT is present in low abundance and is processed to yield two stable introns of 1.5 and 2 kb in addition to at least eight micro (mi)RNAs (Jurak *et al.*, 2010; Umbach *et al.*, 2008, 2009, 2010). The HSV-encoded LATs have multiple functions. Mutants deficient for LAT expression result in increased neuronal death (Thompson & Sawtell, 2001), apoptosis (Perng *et al.*, 2000), enhanced entry into lytic cycle (Chen *et al.*, 1997; Garber *et al.*, 1997) and a reduction in the levels of heterochromatin markers associated with the latent virus genome (Cliffe *et al.*, 2009). These data suggest that the major biological functions of LATs are to promote neuronal survival at an early stage following infection or during reactivation, to negatively regulate viral gene expression via the formation of repressive heterochromatin on lytic promoters and to downregulate expression of viral immediate-early (IE) protein synthesis by LAT-encoded micro RNAs. Such functions would serve to stabilize latency and introduce important checkpoints in the control of reactivation. In addition, there is accumulating evidence from both experimental model systems and examination of human trigeminal ganglia (TG), supporting an important role for virus-specific CD8^+^ T-cells in the surveillance of neurones within latently infected ganglia (reviewed by Sheridan *et al.*, 2007). Such virus-specific CD8^+^ T-cells have been shown to rescue cells at early stages of reactivation through non-cytolytic mechanisms involving secretion of gamma interferon (Liu *et al.*, 2000, 2001) and inactivation of the essential IE ICP4 protein by CD8^+^ T-cell-derived granzyme-B (Knickelbein *et al.*, 2008). The net outcome of such immunological control is a block in progression of a full lytic cycle and stabilization of latency. These data are inconsistent with a simple default model of latency, which is centred on latency establishment being the consequence of a failure of IE gene activation. We have previously utilized a Cre reporter mouse model system to perform a historical analysis of virus promoter activation in the dorsal root ganglia (DRG) (Proença *et al.*, 2008). In this system, virus expressed Cre-recombinase induces a permanent genetic modification in the host cell that results in reporter gene activation. Therefore, if a cell survives to become latently infected it will be marked for life and can be easily identifiable. Using recombinant viruses expressing Cre-recombinase under the control of latent and lytic cycle virus promoters revealed that IE ICP0 promoter (ICP0P) activity could precede latency establishment in approximately 1/3 of the total latent reservoir. IE promoter activation is mediated by the virion transactivator VP16, which although considered a leaky-late (L_1_) gene (Lieu & Wagner, 2000) has recently been shown to be differentially regulated in neurones and to play a central role in the initiation of hyperthermic stress-induced virus reactivation (Thompson *et al.*, 2009). In this paper, we have sought to determine whether VP16 promoter (VP16P) activation can precede latency establishment and therefore provide a mechanistic basis for the observed activation of the ICP0P prior to latency establishment. Using recombinant viruses encoding Cre-recombinase under the control of the L_1_ VP16 or early (E) thymidine kinase (TK) promoters, we show that infection of reporter mice with these viruses results in inefficient cell marking and consequent neuronal reporter gene expression during latency. These data are consistent with the view that expression of these representative E and L_1_ gene products is largely incompatible with cell survival and establishment of latency. In contrast, infection of reporter mice expressing Cre-recombinase under ICP0 or ICP4 IE promoter control results in high frequency cell marking that is maintained during latency. We conclude that a significant proportion of the latent cell reservoir experience VP16-independent IE promoter activation prior to the establishment of latency.

## Results

### *In vitro* and *in vivo* characterization of HSV-1-based recombinants encoding Cre-recombinase

**Fig. 1.**
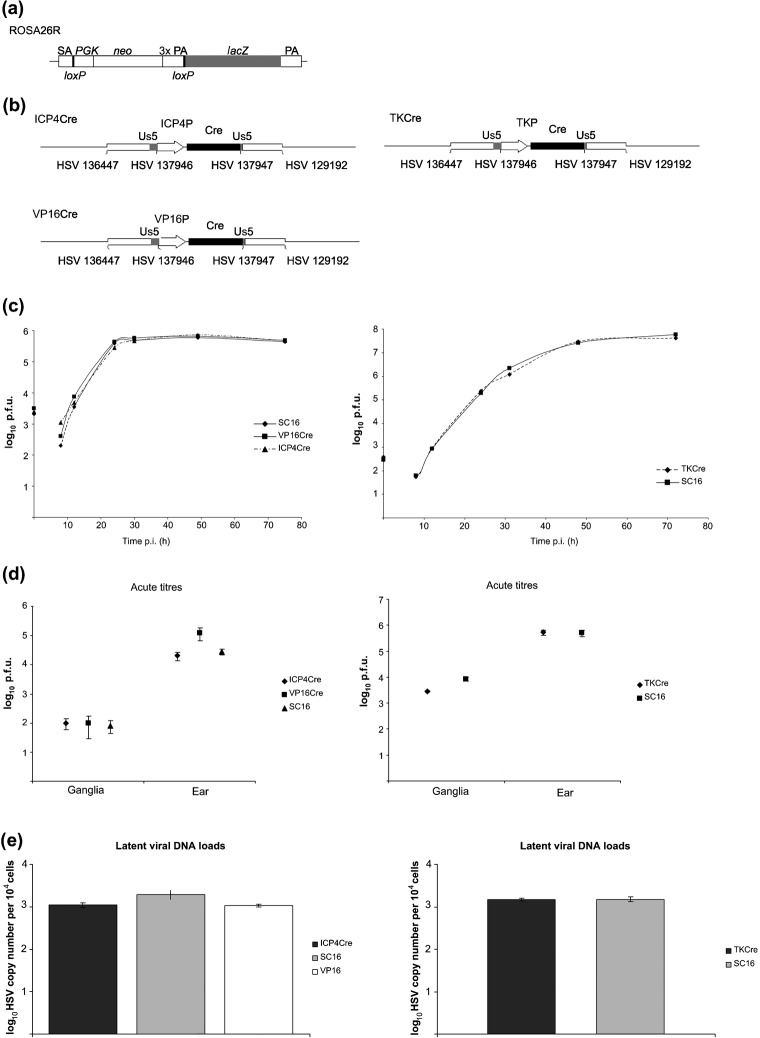
(a) Structure of the ROSA26 locus in R26R reporter mice. The transgene contains a splice acceptor sequence (SA) upstream of a neomycin phosphotransferase gene (*neo*) flanked by *lox*P sites and downstream of a *lacZ* gene. Following Cre recombination, the *neo* gene is removed and the *lacZ* gene is constitutively expressed by the ROSA26 promoter (Soriano, 1999). (b) Genomic structures of Cre-expressing viruses: HSVICP4Cre, HSVTKCre and HSVVP16Cre have a Cre expression cassette inserted in the non-essential Us5 region. This cassette contains the promoter of interest upstream of Cre-recombinase. The Cre gene is fused to a nuclear localization signal and contains an intron 578 bp downstream of the transcription start site. (c) *In vitro* growth curves of recombinants and WT strain SC16 are from a single experiment performed in BHK cells. (d) *In vivo* pathogenicity studies. Virus titres in ears and pooled CII, CIII and CIV ganglia of BALB/c (HSVTKCre) or C57B6 (HSVICP4Cre/VP16Cre) mice sampled at day 5 p.i. Data points represent mean viral titres from five mice±sem for each recombinant and WT strain SC16. Each panel represents an independent experiment. (e) Latent DNA loads of recombinant viruses. Real-time PCR was performed on DNA extracted from latently infected ganglia (CII, CIII and CIV pooled from five mice) using as targets ICP0 and APRT. Values represent the mean±sem of the numbers of the HSV genome copies per 10^4^ copies of APRT from triplicate PCRs.

Viruses expressing Cre-recombinase under IE ICP4, E TK or L_1_ VP16P control were constructed on the HSV-1 strain SC16 background as described in Methods. The genomic structures of all recombinant viruses were confirmed by restriction enzyme digestion and Southern blot hybridization analyses (data not shown). Schematic representations of the ROSA26 locus in the ROSA26 reporter (R26R) animals and recombinant virus structures are shown in Fig. 1(a, b). All recombinants replicated with wild-type (WT) kinetics *in vitro* (Fig. 1c). Acute phase replication in the ears and CII, CIII and CIV sensory ganglia of mice revealed no obvious growth deficit of recombinants in comparison to WT SC16 (Fig. 1d). Real-time PCR-based quantification of latent DNA loads in sensory ganglia revealed that all recombinants established latency to WT levels (Fig. 1e). These data indicate that the recombinants are phenotypically indistinguishable from WT virus *in vivo* and are consistent with previous observations concerning the lack of detectable phenotypes of viruses carrying gene insertions in the Us5 locus (Balan *et al.*, 1994; Proença *et al.*, 2008; Thompson *et al.*, 2003).

### ICP4 promoter activation is compatible with latency establishment in a subpopulation of infected neurones

**Fig. 2.**
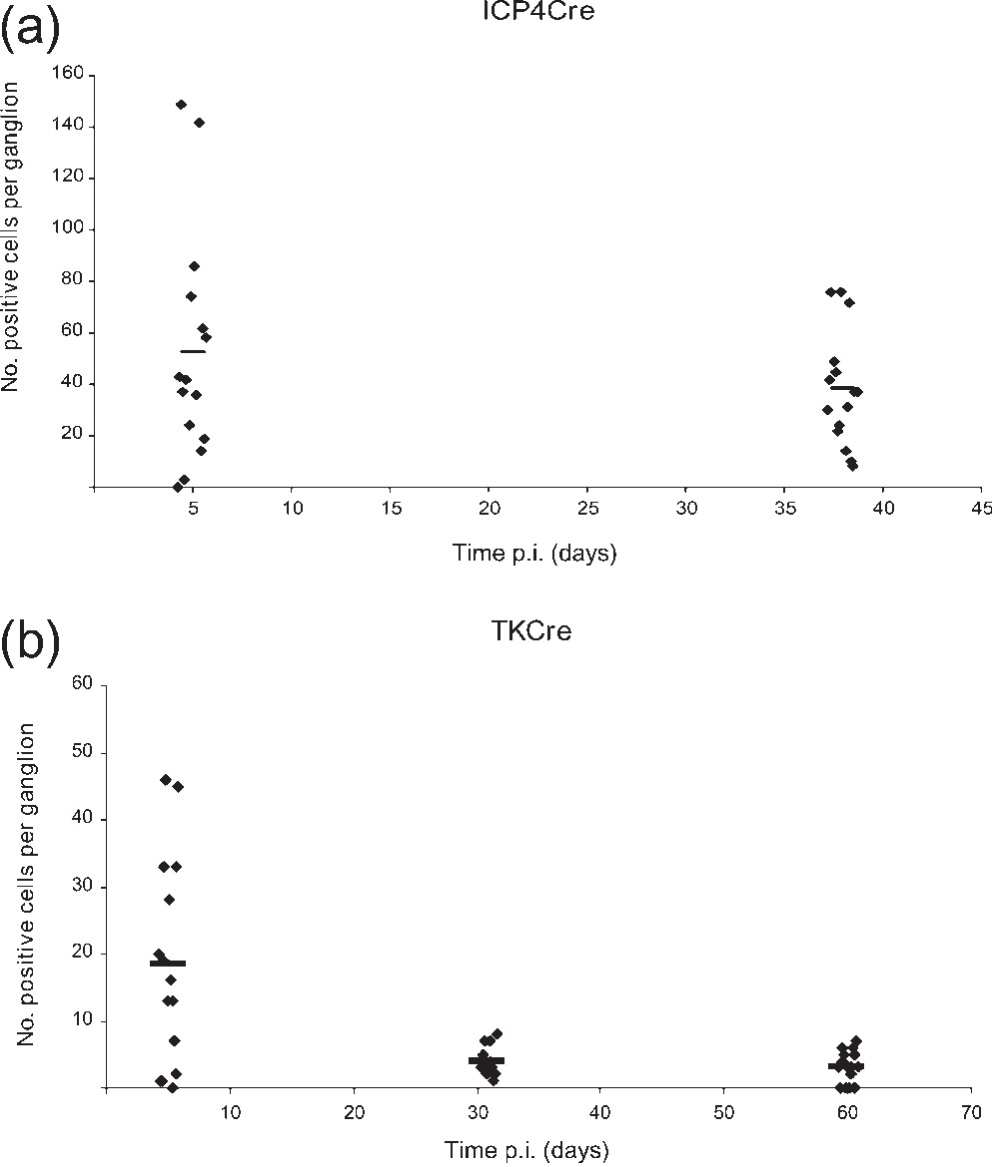
*In vivo* reporter gene expression activated by HSVICP4Cre and HSVTKCre. Number of positive cells per ganglion detected at the specified time points p.i. of R26R mice with HSVICP4Cre (a) and HSVTKCre (b). Each symbol represents an individual ganglion and the bar represents the mean at the given time point.

Previous studies have shown that infection of R26R mice with HSV-1 recombinants expressing Cre-recombinase under the control of the human cytomegalovirus (HCMV) major (M) IE or LAT promoters results in efficient reporter gene activation and marking of latently infected neurones (Proença *et al.*, 2008; Wakim *et al.*, 2008). The observation that Cre-recombinase expressed from the IE ICP0, but not the true late (L_2_) glycoprotein (g)C promoter, results in marking of approximately 1/3 of the total latent cell reservoir indicates that a significant proportion of infected cells experience ICP0P activity prior to the establishment of latency (Proença *et al.*, 2008). In order to determine whether activation of the ICP0P prior to latency establishment is a specific property of this IE promoter or a more general feature of HSV-1 IE promoters we examined the properties of the IE ICP4 promoter (ICP4P). A recombinant virus containing the ICP4P linked to Cre-recombinase (HSVICP4Cre) was constructed and used to infect R26R mice. CII, CIII and CIV DRGs were pooled from five mice at 5 and 30 days post-infection (p.i.), X-Gal stained and then imaged. At day 5 p.i., a mean (±sem) of 52.6±11.6 beta-galactosidase (β-Gal)-positive cells per ganglion was detected, whereas a mean of 38.2±5.8 positive cells per ganglion was detected at 30 days p.i., a time consistent with the establishment of latency (Fig. 2a). This indicates that both IE ICP0 and ICP4P have the capacity for transient activity prior to the establishment of latency.

### Activation of the E TK promoter is largely incompatible with latency establishment

Having established that IE ICP4P activity can be compatible with cell survival and latency establishment, we next sought to determine whether the detection of marked cells during latency would be a feature of the representative E TK promoter (TKP).

R26R mice were infected with HSVTKCre and killed at days 5 (*n* = 5), 31 (*n* = 4) and 60 (*n* = 5) p.i. and the CII, CIII and CIV DRG were dissected and X-Gal stained prior to imaging. The numbers of marked cells per ganglion are plotted in Fig. 2(b). At day 5 p.i. infection, a mean (±sem) of 18.5±4 positive cells per ganglion was detected. This represents a three- to fourfold decrease in the numbers of cells marked by recombinant viruses encoding Cre-recombinase under IE promoter control. Although all the Cre-expressing recombinants have WT replication kinetics both *in vitro* and *in vivo*, the number of cells marked during the acute phase of infection *in vivo* vary depending on the particular recombinant under investigation. The observed variation in the number of marked cells during acute infection is heavily influenced by the kinetic class of promoter used to drive Cre expression. Thus, in earlier studies (Proença *et al.*, 2008) we have shown that expression of Cre-recombinase under the control of the L_2_ gC promoter is unable to efficiently mediate *lacZ* expression *in vitro* following infection of a reporter cell line and similarly resulted in the inefficient labelling of cells in mouse DRGs during acute infection. This is despite the fact that this recombinant exhibited WT growth kinetics both *in vitro* and *in vivo*.

In the context of lytic infection, timing of Cre expression appears to significantly influence the efficiency of cell marking. We consider it highly probable that placing Cre-recombinase under either E or late (L) promoter control results in inefficient Cre-mediated *lacZ* expression during lytic infection as a consequence of virus-induced cytopathic effects severely restricting host-cell gene expression. Under these circumstances Cre-mediated cell marking will under-represent the true numbers of virus-infected cells.

During latency a mean (±sem) of 3.9±0.7 and 3.1±0.6 marked cells was detected at days 31 and 60 p.i. with HSVTKCre, respectively (Fig. 2b). Statistically the marked cell counts recorded at the latent time points are not significantly different from each other (*P* = 0.54). However, the drop in the number of marked cells between day 5 and each of the latent time points is statistically significant (*P* = 0.036, day 31 and *P* = 0.006, day 60). We interpret this decrease in cell marking to represent the death and loss of productively infected cells during the acute stage of infection.

The stable, low level of cell marking observed with the early TKP during latency at days 31 and 60 p.i. is similar to that observed previously with the L_2_ gC promoter. Thus, gC promoter driven Cre expression resulted in a mean (±sem) of 1.9±0.35 and 2±0.36 β-Gal-positive neurones per ganglion at 30 and 124 days p.i., respectively (Proença *et al.*, 2008). Whether these marked cells represent neurones that have survived E or L_2_ HSV promoter activity during the establishment of latency or are the products of abortive reactivation events is currently unknown. However, in comparison to the high frequency of cell marking observed during latency with HSV recombinants expressing Cre-recombinase under the control of IE or LAT promoters (Fig. 2a and Proença *et al.*, 2008), we conclude that E and L HSV-1 promoter activation is largely incompatible with cell survival and the establishment of latency.

### Activation of the VP16P is incompatible with latency establishment

**Fig. 3.**
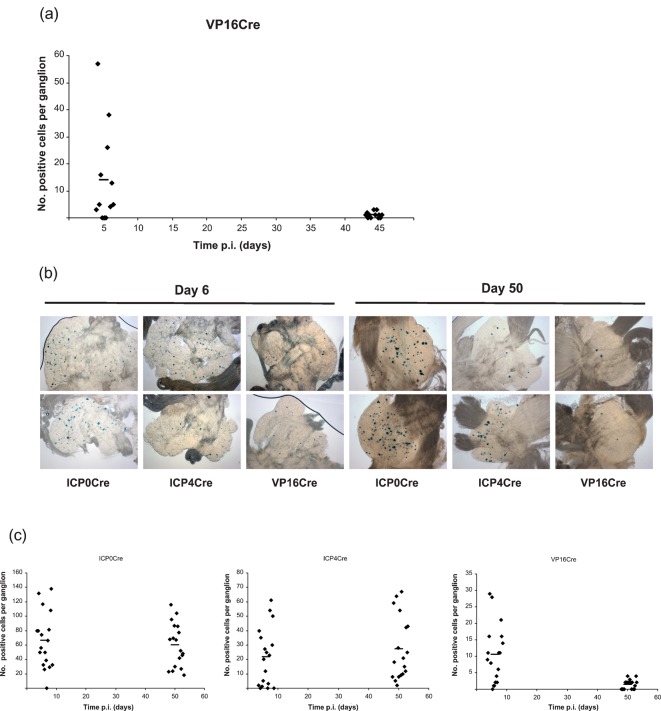
*In vivo* reporter gene expression activated by HSVVP16Cre. (a) Number of positive cells per ganglion detected at the specified time points p.i. of R26R mice with HSVVP16Cre. (b) Light micrographs of X-Gal stained DRG from R26R mice infected with HSVICP0Cre, HSVICP4Cre and HSVVP16Cre at days 6 and 50 p.i. (c) Number of positive cells per ganglion detected following infection of R26R mice with HSVICP0Cre, HSVICP4Cre and HSVVP16Cre at days 6 and 50 p.i. Each symbol represents a ganglion and the bar represents the mean at the given time point.

Recently, the classically defined L_1_ VP16P has been shown to exhibit distinct regulation in neurones, which is functionally important both for acute phase lytic neuronal infection and the earliest stages of reactivation from latency (Thompson *et al.*, 2009). Given that HSV-1 IE gene activation of non-neuronal cells is principally dependent on VP16/Oct-1/HCF complex recognition of IE TAATGARAT motifs (Kristie & Roizman, 1987; Preston *et al.*, 1988; Stern *et al.*, 1989) raises the question of whether the marking of latently infected neurones observed by the IE ICP0 and ICP4P driven Cre constructs is dependent on *de novo* VP16 expression. In this scenario, VP16P activation during latency establishment would be compatible with cell survival in the population of neurones that have been shown to tolerate prior IE promoter activation. With the aim of testing this hypothesis an HSV-1 recombinant encoding Cre-recombinase under the control of the VP16P was constructed and used to infect R26R mice. At days 5 (*n* = 4) and 44 (*n* = 4) p.i., CII, CIII and CIV DRGs were stained with X-Gal and the number of β-Gal-positive cells enumerated (Fig. 3a). At day 5 p.i., a mean (±sem) of 14±5.2 β-Gal-positive cells per ganglion was detected. This low level of cell marking is similar to what we have observed for the TK and gC promoters and therefore appears to be a characteristic feature of promoters exhibiting conventional E and/or L kinetics. By day 44 p.i., the numbers of marked cells detected were significantly lower than at the acute time point and decreased to a mean (±sem) of 1.1±0.3 positive cells per ganglion (*P* = 0.019). The frequency of marked cells during latency was therefore similar to that observed with the gC and TKPs (Fig. 2b and Proença *et al.*, 2008) and in comparison to IE promoter driven Cre constructs we observed a >10-fold decrease in numbers of marked cells identified during latency.

For a more accurate comparison of the frequency of cell marking by virus recombinants encoding Cre-recombinase under either VP16, ICP0 or ICP4P control, R26R mice were infected in parallel with HSVVP16Cre, HSVICP0Cre and HSVICP4Cre. At day 6 p.i., the mean number of β-Gal-positive cells (±sem) detected in CII, CIII and CIV DRG were: 66±9.1, 21±4.7 and 10±2 positive cells per ganglion for ICP0, ICP4 and VP16P driven Cre constructs, respectively. At the latent time point, 50 days p.i., the mean number of β-Gal-positive cells (±sem) detected was 60±7, 27±5 and 1.4±0.32 positive cells per ganglion for the ICP0, ICP4 and VP16P driven Cre constructs, respectively (Fig. 3b, c). Therefore, cell marking of latently infected neurones mediated by IE promoter activation appears to occur in a manner that is not dependent on prior VP16P activation.

**Fig. 4.**
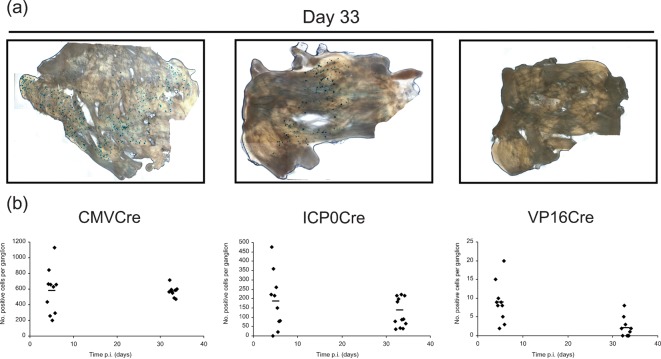
*In vivo* reporter gene expression activated by HSVCMVCre, HSVICP0Cre and HSVVP16Cre in the TG of R26R reporter animals. (a) Light micrographs of representative TG infected with HSVCMVCre, HSVICP0Cre and HSVVP16Cre at 33 days p.i. (b) Number of positive cells per TG detected following infection of R26R mice with HSVCMVCre, HSVICP0Cre and HSVVP16Cre at days 5 and 33 p.i. Each symbol represents a TG and the bar represents the mean at the given time point.

Since previous reports have highlighted differences in the behaviour of HSV-1 in murine DRG versus TG (Sawtell & Thompson, 1992), we next examined the possibility that such anatomical differences could impact on HSV-1-mediated cell marking in the R26R model system. In order to target the TG, reporter animals were infected by scarification of the whisker pad. At 5 and 33 days p.i., TG ganglia were X-Gal stained and imaged (data not shown and Fig. 4a). At day 5 p.i., a mean (±sem) of 577±287, 187±152 and 9±5 β-Gal-positive cells per TG ganglion was detected following infection with HSVCMVCre, HSVICP0Cre and HSVVP16Cre, respectively. At the latent time point, 33 days p.i., a mean (±sem) of 570±68, 136±76 and 2±2.7 β-Gal-positive cells per ganglion was detected for HCMV MIE, ICP0 and VP16P driven Cre constructs, respectively (Fig. 4a, b).

From the data accumulated from both the ear pinna and whisker pad infection models that target DRG and TG, respectively, we conclude that as for the TK and gC promoters expression of Cre-recombinase from the L_1_ VP16P is largely incompatible with cell survival and latency establishment. Therefore, HSV-1 IE promoter activation leading to the marking of latently infected sensory neurones is likely to occur through a VP16-independent mechanism.

### VP16-independent activation of the ICP0 and ICP4 promoters in primary neuronal cultures

**Fig. 5.**
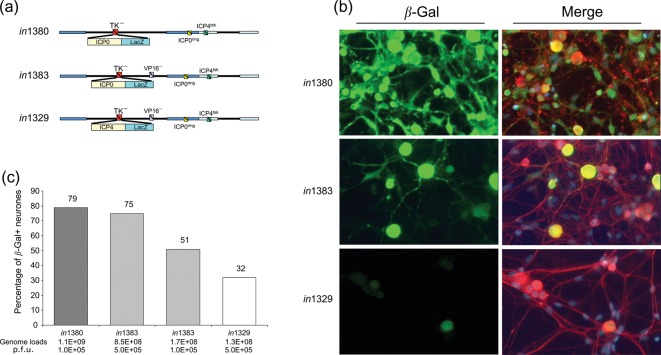
Activation of the ICP0 and ICP4Ps in primary neuronal cultures. (a) Genomic structures of *in*1380, *in*1383 and *in*1329. (b) Representative immunofluorescent images of neuronal cultures infected with 10^5^ p.f.u. of *in*1380 and *in*1383 and 5×10^5^ p.f.u. of *in*1329. Cultures were fixed 2 days p.i. and immunostained for expression of β-Gal (FITC, green) and neurone-specific β-tubulin III (Cy3, red) and counterstained with DAPI to show cell nuclei (blue). The merged images show co-visualization of FITC, Cy3 and DAPI fluorescence in which co-localization of β-Gal and β-tubulin III gives a yellow–orange signal. (c) Summary histogram showing the percentage of β-Gal-positive neurones detected 2 days following infection with *in*1380, *in*1383 and *in*1329. The infection dose in terms of p.f.u. or genome load per well is indicated.

Our studies using reporter mice suggest that marking of latently infected neurones by virus recombinants encoding Cre-recombinase under IE promoter control is likely to reflect neuronal-specific activity of these promoters. In order to examine the potential of these promoters to be activated in neurones in the absence of VP16 we examined the properties of VP16 transactivation-deficient mutants in primary neuronal cultures. DRG cultures were prepared from neonatal rats as described in Arthur *et al.* (2001). All recombinant viruses were based on HSV-1 strain 17 *in*1814 mutant that has a 12 bp insertion within the VP16-coding sequences (Ace *et al.*, 1989). As a consequence of this mutation, *in*1814 exhibits a severe deficit for VP16-mediated IE promoter activation. In order to investigate the VP16 dependence of ICP0P activation following infection of primary neurones we utilized two previously described replication defective virus mutants designated *in*1383 and *in*1380, which contain an ICP0P *lacZ* reporter cassette inserted in the TK locus. *in*1383 has the VP16 mutation of *in*1814, a deletion of the essential ring domain of ICP0 and a temperature-sensitive mutation of ICP4 that inactivates this essential regulatory protein at temperatures >38 °C (Preston *et al.*, 1997). *in*1380 is a VP16 rescuant of *in*1383 (Fig. 5a).

Primary neuronal cultures seeded at a density of 800–1000 neurones per well were infected with either VP16+ (*in*1380) or VP16− (*in*1383) mutants at 10^5^ p.f.u. per well. We estimate that the number of non-neuronal cells per well exceeds the number of neurones plated by a factor of five therefore the theoretical m.o.i. at 10^5^ p.f.u. is approximately 20 p.f.u. per cell. Cultures infected with *in*1380 or *in*1383 analysed 2 days p.i., resulted in readily detectable ICP0P driven β-Gal expression in 79 and 51 % of β-tubulin III-positive cells, respectively (Fig. 5). Analyses of viral genome loads revealed that in this experiment cultures infected with *in*1383 had received 1.7×10^8^ genomes per well, whereas cultures infected with *in*1380 had received 1.1×10^9^ genomes per well (Fig. 5c). Therefore, even following infection with approximately 6.5-fold fewer input genomes VP16-independent activation of the ICP0P was evident in greater than 50 % of neurones. Increasing the *in*1383 virus input to 5×10^5^ p.f.u. per well (8.5×10^8^ input genomes) resulted in β-Gal expression in 75 % of neurones. From these data, we conclude that there is no absolute requirement for VP16-mediated ICP0P activation in cultured sensory neurones and that the efficiency of activation of this promoter is input dose dependent. In contrast, ICP0P activation in β-tubulin III-negative non-neuronal cells, showed a strong VP16 dependence. Thus, following infection of cultures with the VP16-deficient mutant *in*1383 at 5×10^5^ p.f.u., β-Gal expression was detected in 39 % of non-neuronal cells, whereas infection of cultures with an equivalent input of the VP16 rescuant virus (*in*1380) resulted in ICP0P driven β-Gal expression in 89 % of β-tubulin III-negative non-neuronal cells (data not shown).

In order to determine whether the VP16-independent activation of IE ICP0P activity in neurones was a specific feature of this promoter we next examined the properties of the ICP4P. Infection of neuronal cultures with 5×10^5^ p.f.u. (1.3×10^8^ input genomes) per well of the VP16- mutant (*in*1329), containing an ICP4P *lacZ* reporter cassette in the TK locus (Homer *et al.*, 1999), resulted in β-Gal expression in 32 % of neurones and no detectable reporter gene expression in non-neuronal cells. Although, the level of β-Gal expression was notably weaker for the ICP4P than that observed for the ICP0P examined in the context of *in*1383, it appears that at least for the two IE promoters selected for the assay, there appears to be no absolute requirement for VP16 for their activity in cultured sensory neurones. These *in vitro* data, in conjunction with our observations using reporter mice infected with viruses encoding Cre-recombinase under IE or VP16P control indicate the existence of VP16-independent mechanisms of IE promoter activation in sensory neurones.

## Discussion

In this study, we have analysed the extent of viral promoter activation compatible with latency establishment using a previously described Cre reporter mouse system (Proença *et al.*, 2008; Wakim *et al.*, 2008). The system involves the infection of R26R reporter mice with HSV-1 recombinants engineered to express Cre-recombinase under the control of representative IE, E or L virus promoters. In the event of promoter activation, associated with cell survival and the establishment of latency, Cre expression induces a permanent genetic change in the host cell resulting in reporter gene expression and cell marking.

Using this system, we show that IE ICP4P driven Cre expression results in the marking of ganglionic cells during acute and latent infection in a pattern similar to that previously described for the IE ICP0P (Proença *et al.*, 2008). In a direct comparison, 66±9.1 and 21±4.7 positive cells per ganglion were detected 6 days after ear pinna infection with HSVICP0Cre and ICP4Cre recombinants, respectively. Examination of latently infected sensory ganglia 50 days p.i. revealed 60±7 and 27±5 positive cells per ganglion for the HSVICP0Cre and ICP4Cre recombinants, respectively. There is no significant difference between the numbers of marked cells detected at days 6 and 50 for either virus (*P*>0.3). However, when comparing the numbers of marked cell achieved at days 6 or 50 between the two viruses the differences are highly significant (*P*<0.0009) with higher frequency of marking achieved by the ICP0Cre recombinant at both acute and latent time points. Given that HSV IE promoters are largely silenced during latency we conclude that both IE promoters exhibit activity prior to latency establishment, but do so with differing efficiency. The difference in cell marking achieved by the two recombinants is unlikely to be due to different viral replication kinetics since both viruses replicate in a manner indistinguishable to WT strain SC16 *in vivo*, and establish normal latent DNA loads. Since Cre-mediated recombination is a stochastic event, the efficiency of which is influenced by the level and duration of recombinase expression (Nagy, 2000), it seems likely that the different levels of cell marking observed for the IE ICP0 and ICP4P reflect the relative strength of these promoters in neurones. In support of this view, investigation of the activity of the ICP0 and ICP4P in the context of replication defective vectors lacking the transactivating function of VP16, revealed enhanced intensity of reporter gene expression and higher numbers of neurones expressing β-Gal driven from the ICP0 versus the ICP4P. Thus, infection of primary neurones with *in*1383 (ICP0-lacZ) resulted in strong β-Gal expression in 51 % of neurones, whereas *in*1329 (ICP4-LacZ) resulted in lower levels of reporter gene expression in 32 % of neurones. Our experiments with Cre reporter animals reveal a similar disparity in the efficiency of cell marking with the ICP0P, resulting in Cre-mediated reporter gene activation in over twice as many neurones as the ICP4P.

**Fig. 6.**
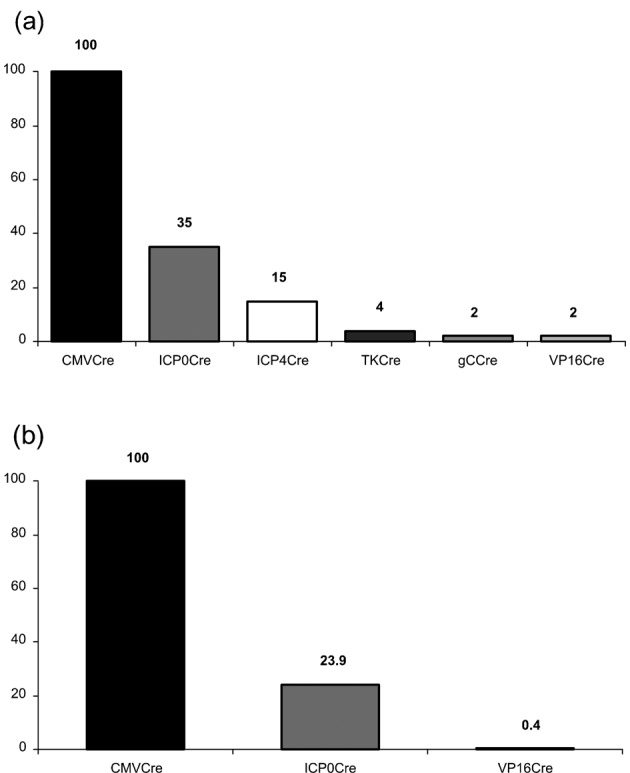
Relative numbers of marked cells detected during latency following infection with the specified Cre-expressing HSV recombinants in DRG (a) and TG (b) of R26R reporter animals.

In contrast to the data obtained with recombinant viruses expressing Cre under IE promoter control a recombinant engineered to express Cre under the control of the E TKP did not mark significant numbers of neurones (Fig. 2a). The frequency of cell marking was similar to that previously described for the L_2_ gC promoter (Proença *et al.*, 2008). Of particular interest was the observation that HSVVP16Cre behaved in a similar fashion to the TK and gC Cre recombinants. Thus, recombinant viruses expressing Cre-recombinase from either E, L_1_ and L_2_ promoters resulted in inefficient cell marking during acute infection and resulted in less than four marked neurones per ganglion during latency. As summarized in Fig. 6, a similar pattern of cell marking was observed both in DRG and TG neurones, indicating that virus promoter activation is not significantly influenced by either ganglionic type or anatomical site of infection.

*De novo* expression of VP16 at early times following neuronal infection has been shown to be critical for entry into lytic cycle (Thompson *et al.*, 2009) and would be consistent with the observed low frequency of cell marking by the HSVVP16Cre recombinant during latency. Thus, neurones exhibiting VP16P activity would initiate the lytic programme of gene expression and enter the productive cycle, which would result in cell death. Less clear is the inability of the reporter mouse system to reveal VP16P Cre marked cells at early times following acute infection. An expectation of neuronal-specific *de novo* activation of the VP16P is that it would express Cre-recombinase with similar kinetics to the IE ICP0 and ICP4P and therefore result in efficient cell marking during acute ganglionic infection.

By comparison of the numbers of marked cells generated by the IE promoters and the VP16P we conclude that IE promoter activation compatible with neuronal cell survival and latency establishment is likely to be independent of *de novo* synthesis of VP16. It has previously been shown that VP16 is not required for latency establishment *in vivo* (Ecob-Prince *et al.*, 1993; Marshall *et al.*, 2000; Steiner *et al.*, 1990). In contrast, *de novo* synthesis of VP16 has a key role in the initiation of virus gene expression in neurones. Thus, neuronal-specific activation of VP16 during acute infection and hyperthermia-induced reactivation is associated with lytic cycle entry and productive cycle replication (Thompson *et al.*, 2009), events that would preclude survival of neurones experiencing VP16P activation. Our inability to efficiently mark latently infected neurones with HSVVP16Cre is therefore consistent with the view that *de novo* activation of the VP16P is incompatible with cell survival and latency.

At present, we cannot formally exclude the possibility that our inability to detect efficient cell marking during acute ganglionic infection with the VP16 Cre recombinant is a consequence of transient low level *de novo* expression of Cre-recombinase from this promoter, resulting in inefficient *lox*P-mediated recombination and reporter gene activation. However, our studies using a primary neuronal cell culture system have revealed that IE ICP0 and ICP4P activation in neurones can occur in the absence of functional VP16. It should, however, be noted that these *in vitro* experiments were performed at high multiplicities of infection and high virus DNA input loads. We therefore cannot formally exclude the possibility that the VP16 requirement for IE promoter activation might be greater in neurones at low multiplicities of infection. Nonetheless, our results are consistent with studies using reporter transgenic mice that have shown expression of reporter genes in trigeminal neurones linked to the IE ICP0 and ICP4P, but not the ETK or L_2_ gC promoters (Loiacono *et al.*, 2002, 2003, 2004). Together these data are strongly supportive of the view that HSV IE promoters and in particular the ICP0P can be targeted for activation in sensory neurones via the action of cellular factors.

The biological significance of the population of neurones marked as a consequence of ICP0 or ICP4P activity is unclear. At present, we do not know whether the promoter activity mediating cell marking is indicative of IE protein expression. In the case of ICP0, previous studies have reported the detection of low levels of largely unspliced ICP0 transcripts during latency (Chen *et al.*, 2002; Maillet *et al.*, 2006; Thompson & Sawtell, 2006), suggesting that transcriptional activity is unlikely to result in functional ICP0 protein expression and may reflect a splicing related mechanism of latency maintenance. Of possible significance are the small but highly reproducible numbers of neurones that have experienced and survived E and/or L promoter activation. Given that only a small proportion of cells amongst the pool of latently infected neurones are known to respond to either *in vivo* or *ex vivo* reactivation stimuli it will be of particular interest to examine these subsets of cells and determine whether they represent a reactivation primed population of neurones.

## Methods

### 

#### Cells and viruses.

All recombinant viruses were derived from HSV-1 strains SC16 (Hill *et al.*, 1975) and 17syn+ (Brown *et al.*, 1973).

Viruses were propagated and assayed on BHK cells unless stated otherwise. Cells were grown in Glasgow’s modified Eagles medium supplemented with 10 % FCS and 10 % tryptose phosphate broth. SUA cells are a Vero-derived cell line containing a *lox*P-flanked cassette between the CAG promoter and reporter gene. Cre-recombinase mediates removal of the *lox*P-flanked cassette and expression of *lacZ* (Rinaldi *et al.*, 1999).

The replication defective viruses used for infection of primary neuronal cultures have been described previously (Homer *et al.*, 1999; Marshall *et al.*, 2000; Preston *et al.*, 1997).

#### Plasmids.

All Cre-expressing plasmids were tested in SUA Cre reporter cells by transfection. The construction of all plasmids is described in the Supplementary Methods (available in JGV Online).

#### Construction of recombinant viruses.

BE8 is an HSV-1 strain SC16 recombinant containing a CMV promoter lacZ cassette inserted into the non-essential Us5 locus.

HSVTKCre was constructed by co-transfection of pHD5-TKCre linearized with *Sca*I and BE8-infected cell DNA. HSVTKCre contains the TK Cre-recombinase cassette inserted into Us5 locus at the *Sst*I restriction site at genomic coordinate 137946. The ability of the recombinant to express functional Cre-recombinase was confirmed in SUA cells (data not shown).

HSVVP16Cre was generated by co-transfection of *Pst*I linearized pHD5-VP16Cre and BE8-infected cell DNA placing the Cre-recombinase gene under VP16P control at the non-essential Us5 locus. Cre expression was confirmed by RT real-time PCR (data not shown).

HSVICP4Cre was constructed by co-transfection of *Pst*I linearized pHD5-ICP4Cre and BE8-infected cell DNA placing the Cre-recombinase gene under ICP4P control at the non-essential Us5 locus. Cre expression was confirmed by infection of SUA Cre reporter cell line (data not shown).

Three days post-transfection, infected BHK cell monolayers were harvested, sonicated and recombinant progeny were selected based on failure to stain positive for β-Gal (HSVTKCre, HSVICP4Cre and HSVVP16Cre) due to replacement of the lacZ expression cassette in the parental virus BE8. Viral genomic structures (Fig. 1) were confirmed by restriction endonuclease digestion and Southern blot hybridization analyses (data not shown).

#### *In vitro* growth curves.

*In vitro* assays were performed as described previously (Proença *et al.*, 2008).

#### *In vivo* assays.

*In vivo* replication assays were performed using 7- to 8-week-old female BALB/c or C57B6 mice as described previously (Proença *et al.*, 2008).

R26R reporter mice (Soriano, 1999) were used for the *in vivo* characterization of HSV recombinants encoding Cre-recombinase. Groups of adult mice (>8 weeks of age) that differed in age by less than 12 days were infected with 10^6^ or 2×10^6^ p.f.u. of virus by scarification of the left ear, both ears or both whisker pads. Animal gender was matched per experiment or in the case of experiments including more than one virus, matched per time point. At various times p.i., mice were killed and CII, CIII and CIV cervical ganglia or both TGs were pooled, fixed on ice for 1 h in 4 % paraformaldehyde and stained histochemically for X-Gal as described previously (Lachmann & Efstathiou, 1997).

#### DNA extraction for quantitative real-time PCR.

DNA extractions were performed as described previously (Coleman *et al.*, 2008; Proença *et al.*, 2008).

#### Statistical analysis.

Statistical differences between the numbers of marked cells/sensory ganglia from mice sampled at different time points were determined by the Mann–Whitney test.

#### Primary neuronal cultures.

Primary neuronal cell cultures were prepared as described previously (Arthur *et al.*, 2001).

#### Detection and quantification of reporter gene expression in neuronal cultures.

Neuronal cultures staining and imaging was performed as described previously (Arthur *et al.*, 2001).
